# Analysis of the Statistical Comparability of the Hardness and Wear of Polymeric Materials for Orthodontic Applications

**DOI:** 10.3390/ma14112925

**Published:** 2021-05-28

**Authors:** Ivo Domagała, Krzysztof Przystupa, Marcel Firlej, Daniel Pieniak, Leszek Gil, Anna Borucka, Ireneusz Naworol, Barbara Biedziak, Mariana Levkiv

**Affiliations:** 1 Department of Craniofacial Anomalies, University of Medical Sciences, 70 Bukowska Street, 60-812 Poznań, Poland; ivo.m.domagala@gmail.com (I.D.); marcel-firlej@wp.pl (M.F.); biedziak@ump.edu.pl (B.B.); 2 Department of Automation, Lublin University of Technology, 36 Nadbystrzycka Street, 20-618 Lublin, Poland; 3 Department of Mechanics and Machine Building, University of Economics and Innovations in Lublin, 4 Projektowa Street, 20-209 Lublin, Poland; daniel.pieniak@wsei.lublin.pl (D.P.); leszek.gil@wsei.lublin.pl (L.G.); 4 Faculty of Security, Logistics and Management, Military University of Technology, 2 gen. S. Kaliskiego Street, 00-908 Warsaw, Poland; anna.borucka@wat.edu.pl; 5 Faculty of Safety Engineering and Civil Protection, The Main School of Fire Service, 52/54 Slowackiego Street, 01-629 Warsaw, Poland; inaworol@sgsp.edu.pl; 6 Department of Dental Therapy, I.Horbachevsky Ternopil National Medical University, 1 Maidan Voli, 46000 Ternopil, Ukraine; levkiv@tdmu.edu.ua

**Keywords:** microhardness, scratch resistance, sliding wear, thermocycling, dental biomaterials

## Abstract

Background: Clinical success depends on the contact strength and wear resistance of medical devices made of polymer materials. The scientific goal resulted from the problem of using different methods of surface evaluation of materials used in the production of orthodontic appliances. The purpose of the work was an experimental comparative assessment of indentation hardness and scratch hardness and the sliding wear of four selected polymeric materials used in the manufacture of orthodontic appliances. Methods: Four commercial materials were compared. Shore hardness tests and a scratch test with a Rockwell indenter were performed. A sliding wear test was performed using the ball-on-disc method. Statistical PCA and correlation analyses were performed. Results: The results of scratch hardness measurements using a contact profilometer correlated with the Shore hardness to a greater extent than measurements made using an optical microscope. PCA showed that Shore hardness explains 45% of the total variance in all the results across the materials. Conclusions: The scratch hardness method allows for a more explicit ranking of orthodontic polymeric materials when measurements are made with a profilometer. The ranking of sliding wear resistance should be made separately.

## 1. Introduction

A significant number of medical devices’ parts are nowadays manufactured from polymeric materials [1]. Polymers are used both as coating materials and for components made entirely of this material [2,3]. Currently, many researchers deal with the useful properties of polymer medical devices [4]. The studies of functional properties determining the operational quality, i.e., strength, durability, and reliability of polymer-based medical devices, are of practical significance [5,6,7,8,9]. The degradation of the surface layer of medical devices due to deformation and abrasive wear is one of the factors limiting clinical usefulness [10,11,12,13,14].

Poly(methyl methacrylate) (PMMA) has found wide use in the healthcare field. It is used in orthopedics, prosthodontic dentistry, and many other medical devices [15,16]. Due to the physical and mechanical properties of PMMA, it is often used. PMMA has good scratch and UV resistance, has a relatively high modulus of elasticity and hardness [17]. In addition, in orthodontic applications, it is characterized by low moisture absorption, e.g., of saliva, in the oral cavity [18]. PMMA is also used to produce orthodontic appliances, including biomechanical ones, for the treatment of malocclusion.

Orthodontic splints made of PMMA are used in the treatment of bruxism and pathological thegosis.

Bruxism is a cranio-mandibular dysfunction that is defined as a total parafunction of muscle activity, during the daytime and at night. It is manifested by grinding, tapping, or clenching of teeth. Thus, it has a detrimental effect on the quality of life. The prevalence of daytime bruxism in adults is reported to range from 22.1% to 31%, while sleep bruxism is manifested at 13% [19]. Daytime bruxism affects females more frequently than males, while in sleep bruxism, males are equally affected as females, but this disorder has a tendency to decrease with age [20,21].

**Figure 1:**
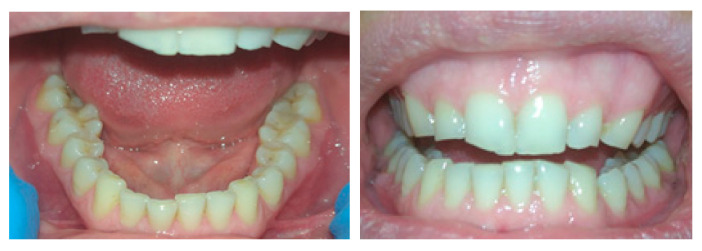
A patient 35 years old with bruxism. Tooth wear with attrition facets.

In the case of bruxism, the tooth enamel wears off, teeth gradually deteriorate, and constant stress on the jaw joint causes inflammation and deformation (Figure 1). This condition occurs, among others, in people exposed to stress. In the air force of one of South American armies, bruxism occurred in up to 30.4% of the military crew [22]. In [23], it was found that bruxism resulted in 22 peak occlusal force measurements ranging from 50 to 200 N per night (7 h of sleep). These forces were measured using properly scaled piezoresistive sensing elements encapsulated in an orthodontic splint [23].

Management of this disease is usually directed toward reduction of stress, relief of muscles spasm and pain, and tooth or restoration protection. The purpose of occlusal splints is to protect teeth and restorations from attrition and adverse traumatic loading. Depending on their designs, occlusal splints can also unload, stabilize, and improve the functions of the temporomandibular joint as well as reduce abnormal muscle activity, reduce muscle pain, and improve functions of the masticatory motor system. It is known that at the time of a spasm, the jaw joint experiences a load several times greater than the pressure on the teeth and joint during mastication of solid food. Therefore, patients with bruxism often complain of severe joint pain in the lower jaw area.

**Figure 2:**
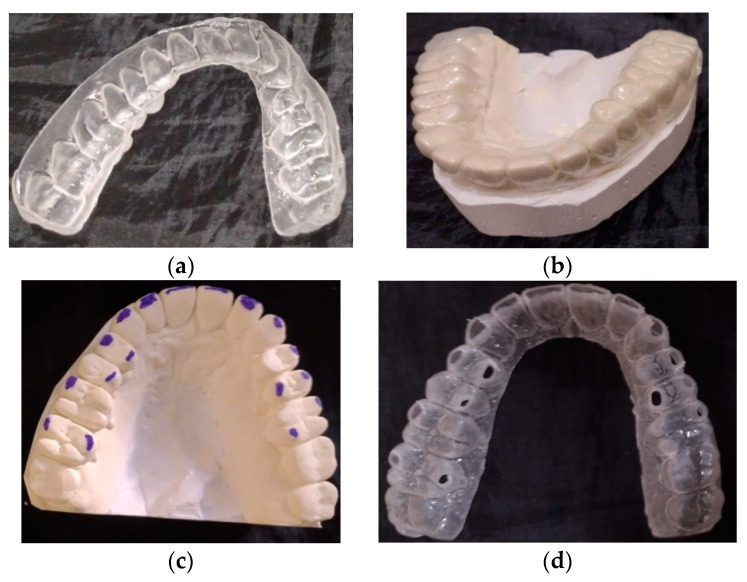
(**a**) Occlusal splint for the treatment of bruxism; (**b**) Splint placed on the model; (**c**) Model with marking of places where the splint damage occurs; (**d**) Worn splint during bruxism treatment.

Figure 2 presents a model of teeth with supercontacts and an occlusal splint with a destroyed surface after usage during one night; the thickness of the material was 2 mm. The damage was probably caused by the material’s low resistance to contact loads. Depending on the worn surfaces on the splint, the dentist can determine the localization of supercontacts (blue color). The picture clearly shows of supercontacts, which can be photographed and used both for diagnosis and for medical purposes for the selection of orthopedic appliance design and dispensary supervision over the quality of treatment.

The researchers found that in places of contact with human teeth, deformations of polymer orthodontic appliances and attrition facets were observed [24].

Mouthguards for patients with bruxism help to avoid fractures of orthodontic appliances during nocturnal attacks of bruxism. The degree of damage of orthodontic appliances is correlated with maximum muscle activity during sleep [24]. During dreaming, the patient clenches their teeth so hard that it can cause failure of dentures or other prosthetic appliances. An occlusal splint prevents tooth displacement, especially in chronic bruxism, when the teeth begin to shift from constant friction in the dentition. The mouthguard/occlusal splint securely fixes them in the gums and does not allow them to move during the load.

For the occlusal splint/mouthguard to be ideal for the patient, it must be made individually. For the manufacture of occlusal splints, a special plastic is used, which is polymerized by pressure. The dentist takes impressions of the patient’s upper and lower jaws, while the technician takes care of the fabrication.

The main purpose of the mouthguard is to protect the patient’s teeth from damage. During a night spasm of the facial muscles, an occlusal splint is placed. Whether the case is of awake or sleep bruxism, occlusal splints should be in the oral cavity during the daytime and at night. For this reason, the splints should be invisible to others, not interfere with speaking or eating, and not feel like a foreign body in the mouth.

Due to that fact that orthodontic appliances are mainly used in advanced cases of bruxism, the mechanical strength and wear resistance of the materials used in the manufacture of the appliances are of great importance. All materials, including teeth tissues, are subjected to abrasion, and therefore, the results of comparative studies of wear resistance of different materials are of particular value.

The hardness of a polymer material is a measure of resistance to concentrated contact forces. Hardness is a measure of the surface resistance of a material to local deformations. It is one of the most important material properties from both a structural and a technological point of view [25]. Static hardness tests are one of the most common tests of the mechanical strength of biomedical materials [8,11,25,26,27,28]. Contact forces can arise in orthodontic treatment of involuntary teeth clenching. Maximum occlusal forces in patients with bruxism range from 450 to 650 N [29]. Mean forces can be 120 N [30] or 380 N [31]. Such concentrated biomechanical forces cause permanent deformation of orthodontic appliance materials at their points of impact. An example is the materials of fangs. In patients with bruxism treated with orthodontic appliances, the surface of the orthodontic appliances is subject to wear. The behavior of polymers under contact forces is different from that of metals. Polymer materials are viscoelastic; in addition, the properties of polymers change with time differently than in the case of metals [32]. One of the experimental methods of assessing the wear resistance of polymers is scratch resistance tests [33]. In a clinical situation, pathological chipping of the enamel on the incisal edge of the incisors leads to sharp edges on the edges of the chewing surfaces of the teeth. This is also the result of inaccurate treatment of the enamel surface and the edges of dental fillings. This condition contributes to the appearance of inequalities along the contour of the filling [34]. In experimental studies, incomparability of indentation hardness and scratch hardness results was noticed [35]. A possible reason is a different method of measuring and determining hardness. The indentation hardness, including Shore’s hardness, depends on the depth of the hollow of the needle of a certain shape and dimensions, which is loaded with a specific normal force to the surface of the sample [36]. In the scratch test, the penetration depth is determined by the contact length between the leading edge of the indenter tip and the material indented [37]. It is influenced by the hardness, modulus, and wear resistance of the material being scratched [38]. Scratch hardness depends largely on the crack width, which is usually determined by means of an optical microscope coupled with a scratch test device. Visual assessment of the features is complicated and may be ambiguous. Many factors, including ambient light conditions, observation angle, color of the sample, and the inspector’s visual acuity, can significantly bias the observation [39]. It is possible that the non-optical method is more suitable for assessing scratches on the surface of polymer materials. In a number of works, researchers have used indentation and scratch methods to evaluate polymer materials. It is therefore important to investigate whether the disagreement between indentation and scratch measurement methods results in differences in the hardness ranking of polymer materials.

In light of the above considerations, the purpose of this work was an experimental comparative assessment of indentation hardness and the wear of four selected polymeric materials used in the manufacture of orthodontic appliances. The hardness rankings of the tested materials was obtained using different hardness methods, and the correlations between the values obtained by the scratch and sliding wear methods were compared.

## 2. Materials and Methods

### 2.1. Materials

The specimens used in the research were made of poly(methyl methacrylate) (PMMA)-based materials. These were four materials from four producers:

1. NextDent Ortho Rigid material (Vertex-Dental B.V., Zeist, the Netherlands; batch no. XK445N01). Material composition given by the manufacturer: methacrylic oligomers, phospine oxides, colorants, and pigments. This material was designated 1A.
2. Erkocryl (ERKODENT Erich Kopp GmbH, Pfalzgrafenweiler, Germany; batch no. 11198). Material composition given by the manufacturer: olymethylmethacrlat. This material was designated 2A.
3. Vertex Orthoplast (Vertex-Dental B.V., Zeist, the Netherlands; batch no. XH212P05), blue. Material composition given by the manufacturer: methyl methacrylate, ethylenglycol dimethacrylate N,N-dimethyl-p-toluidine. This material was designated 3A.
4. Material with the same name and composition as 3A but orange; in the article, marked 4A (Vertex-Dental B.V., Zeist, The Netherlands; batch no. XH153L03).

Flat specimens with thickness ranging from 2.5 to 6 mm were developed. Thicker specimens were used in Shore hardness tests and thinner samples in scratch hardness and wear tests. The specimens were polished with abrasive discs (granulation P600, P1200, P2400, and synthetic polishing pad) using a Saphir 550 single-wheel grinder and polisher (ATM Gmbh, Mammelzen, Germany) and then cleaned in water. The specimens were aged in an artificial saliva (in accordance with the technical standard ISO 10271:2012; pH = 5.3) bath at 37 ± 1 °C for 48 h in a Q-Cell temperature chamber (Pol-Lab, Wilkowice, Poland). Five specimens were made of each type of material for the hardness test and the same number for the scratch resistance and wear tests.

### 2.2. Shore Hardness Test

Shore hardness measurements were performed. This method involves measuring the penetration depth of a steel indenter with a conical tip into the sample surface. Tests were carried out on a Shore HPE II durometer (Bareiss Prüfgerätebau GmbH, Oberdischingen, Germany). A hardness indentation and conversion device were attached to a BS 61 II support (Bareiss Prüfgerätebau GmbH, Oberdischingen, Germany). Due to the properties of the tested samples, the indentation hardness was tested on the Shore D scale (scale from 0 to 100 Shore degrees). The device and the test met the requirements of the technical standard ISO 868 [36]. The dimensions of the samples were also adjusted to these requirements, and samples with a thickness of at least 6 mm were used [36]. The test load of the indenter in the Shore D method was 44,450 mN (5000 g). The measure of hardness is a value inversely proportional to the size of the cavity created under the action of 44,450 mN [37]. The indentation hardness is dependent on the viscoelastic behavior of the polymeric material. It is also related to the surface elasticity modulus. It is possible to calculate the modulus of elasticity based on measurements according to the Shore A scale [40].

### 2.3. Scratch Resistance Test

**Figure 3:**
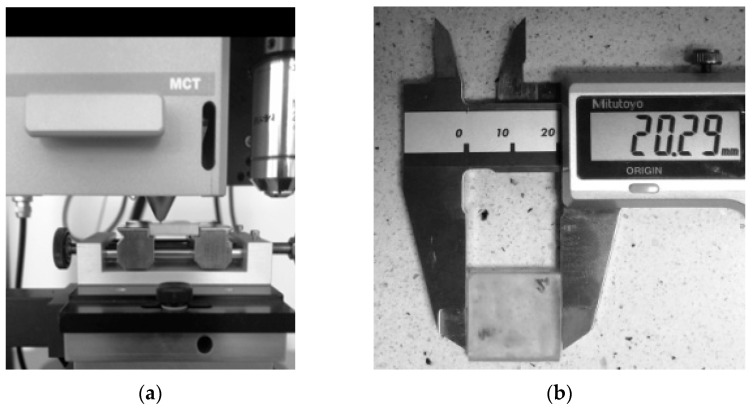

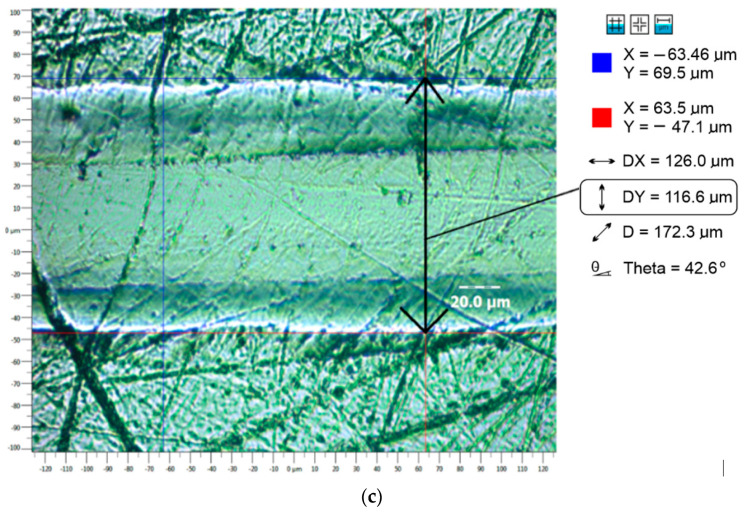
Scratch test and samples used in the test: (**a**) sample in an MCT holder for scratch test; (**b**) the width of sample used in the scratch test; (**c**) and scratch width measurement (SW_mic_) using an optical microscope.

Samples with a thickness in the range of approx. 2.5–3 mm (Figure 3a) were tested on a Micro Scratch Tester (MST, Anton Paar GmbH, Graz, Austria). These dimensions were consistent with the MCT specimen holder (Figure 3a). One of the surfaces of rectangular specimens with nominal dimensions of 20 × 20 mm^2^ (Figure 3b) was processed on a laboratory grinding-polisher. On this surface, scratches were made with a Rockwell indenter in the form of a diamond cone with a rounding radius of 100 µm. The test load, the vector of which was perpendicular to the tested surface, was 2 N (Fn). The scratch test was run at a speed of 3 mm/min. The scratch length was 2 mm. The shape and geometrical dimensions of the scratch were assessed microscopically (Figure 3c). The microscope was an integral component of the MCT device.

**Figure 4:**
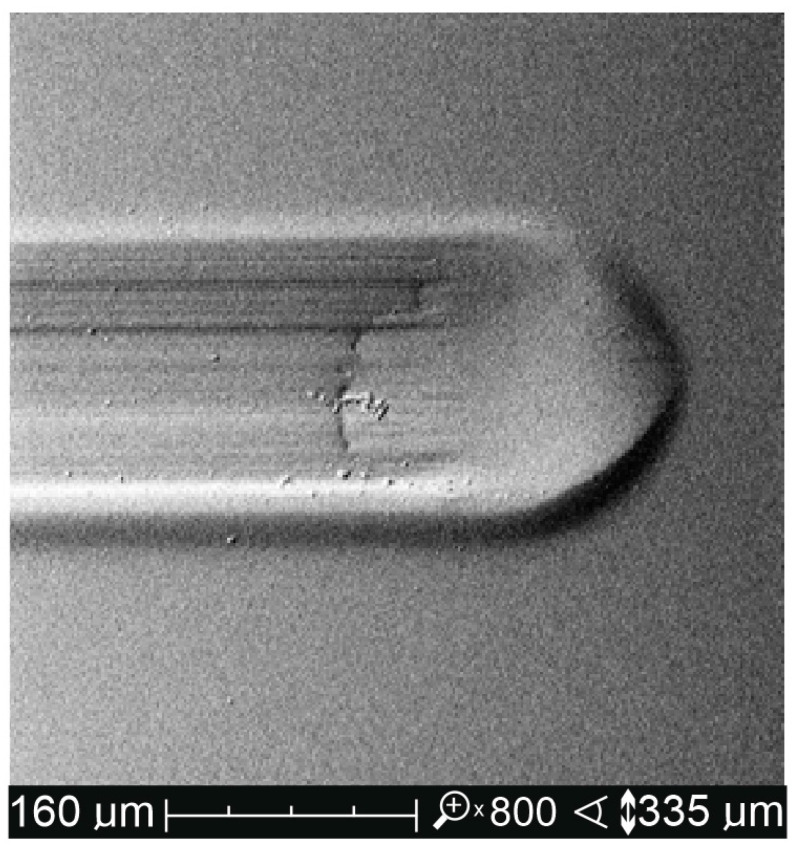
SEM image of topographic pile-up of the polymer material.

**Figure 5:**
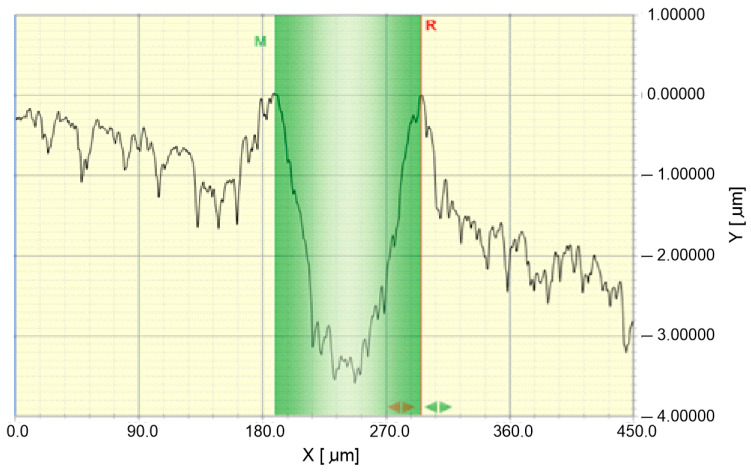
Measurement of the gap width (SW_profil_) as the distance between the highest points of plastic pile-up (width of the green field) and the cross-sectional area of the gap, S_ar_.

The crack geometry was then tested using a Dektak 150 (Veeco, Plainview, NY, USA) contact profilometer. The profilometer allows 2D topography and 3D surface measurements with a resolution of 0.01 µm in the Z axis. It is equipped with 2 measuring tips (interchangeable stylus) with a rounding radius of 2 and 12.5 μm. In this research, a stylus with a rounding radius of 2 μm was used, to which an axial force of 3 mg was applied. The measurement resolution was set at 0.1 µm. The measuring path was in the range of 500–1000 µm. The use of a profilometer made it possible to reveal the nature of the material deformation. The formation of scratches is associated with the formation of permanent plastic deformations not only at the bottom of the furrow but also at its side edges, in the form of a plastic pile-up (Figure 4). Scientific papers on the scratch resistance of polymer materials, including [35,38,39], state that the width of the crack should be measured taking into account the plastic heights on the edges of the furrow, which is not possible to determine using an optical microscope, i.e., it is not possible to determine the highest points of plastic elevations (Figure 5). To better illustrate the size of the surface damage of the tested materials, the *S_ar_* furrow cross-sectional area was also measured. The horizontal axis 0 was plotted through the highest points of plastic heights. Vertical lines limiting the M and R measuring range also passed through these points. Consequently, the surface in the area bounded by the 0 axis was measured by the M and R lines and the surface profile. It is worth noting that in [35,38,39,40,41,42], the *S_ar_* parameter was not taken into account in the assessment of scratches, although it is often used in works in the field of tribology [43].

Based on the obtained results of the crack width tests using the optical microscope and the contact profilometer, the scratch hardness *Hs* was determined. The formula presented in [44] was used to calculate *Hs*:

(1) $$Hs=\frac{4\cdot x\cdot Fn}{\pi \cdot SW^{2}}$$

where:

*Hs*—scratch hardness in N/mm^2^;
*Fn*—normal force in N;
*SW*—joint width in mm; and
*x*—parameter accepted in the range of 1 ÷ 2, the value of 1 adopted as per [45].

### 2.4. Sliding Wear Test

**Figure 6:**
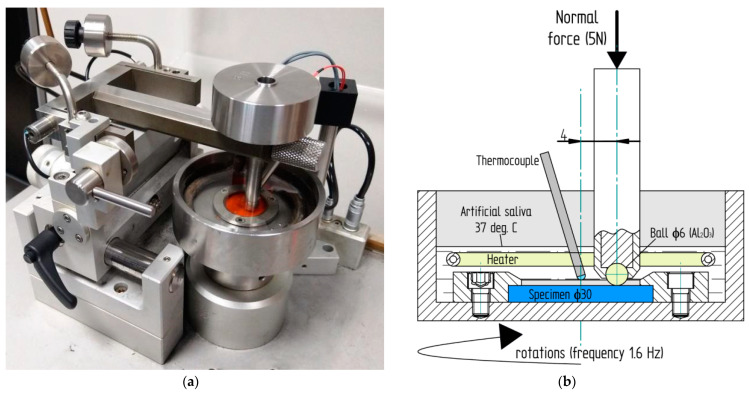
Ball-on-disc wear-testing machine (**a**) and schematic representation (cross section) of sliding wear test (**b**).

Sliding friction studies were conducted using a universal microtribometer (CSM Instruments SA, Neuchâtel, Switzerland) (Figure 6a) in a ball-on-disc configuration (Figure 6b). It is equipped with a temperature-measuring system based on the thermocouple, which is proven to be one of the best temperature sensors used under similar conditions [46,47,48]. Specimens were tested in artificial saliva at a constant temperature of 37 °C. As discs, cylindrical specimens made of polymer materials were used (the thickness of the samples was 2.5–3 mm, and the diameter was 30 mm), while the Ø 6 mm counter specimens—balls—were made of alumina (Al_2_O_3_). The material and diameter of the counter specimens were selected based on our previous experience [49,50]. During the test, the ball was immobile, while the disc rotated with 1.6 Hz frequency (rotating speed 90 rpm). A constant load of 5 N was applied (Figure 6a).

### 2.5. Statistical Analysis

The statistical analysis aimed to evaluate the individual measurement results in the analyzed groups of materials and the relationships between them. It was made according to the following algorithm.

Basic descriptive statistics were determined first for general evaluation. Then, the distribution of the examined feature in the group was assessed. The assumption of normal distribution was analyzed using the Shapiro-Wilk test (due to the small number of observations in the groups; *n* = 5). In the next step, the homoscedasticity of variance was tested using the Levene test.

In the case of empirical distributions following a normal distribution and when the homogeneity of variance was confirmed to clarify whether the type of material could be the reason for differences between the observed measurement results, analysis of variance (ANOVA) was used. This is the statistical method that allows us to test whether an independent variable (factor) affects the results of a dependent variable (univariate analysis of variance) or assess the effect of multiple independent variables (factors) on the value of the dependent variable under consideration (multivariate analysis of variance). In the study presented herein, the analysis of variance was used to verify the hypothesis of equality of variance in populations defined by the material variable. If the assumptions about the normality of the distribution and homogeneity of variance were not met, the non-parametric Kruskal-Wallis test was used.

In the next stage of the study, principal component analysis (PCA) was used. This method involves the determination of entirely new variables (principal components) that are linear combinations of observed (original) variables. The rotation of the coordinate system in such a way to maximize the variance of the first and then of subsequent coordinates is taken as the main objective of this method. PCA allows for the classification of data, especially multidimensional ones, where the reduction of variables results in feature extraction in the form of obtained principal results [51,52,53].

Before starting principal component analysis, it is necessary to check the basic assumption of PCA, i.e., the correlation of variables. The higher the correlations between the original variables, the more justified the use of this analysis [52,53]. The Bartlett test was used to check this. Moreover, it is then necessary to calculate the Kaiser-Mayer-Olkin (KMO) coefficient, which checks the degree of correlation of the primary variables, i.e., the strength of the evidence in favor of the meaningfulness of conducting principal component analysis. This coefficient takes values in the range <0, 1>. The assumptions are considered to be satisfied when the KMO coefficient takes a minimum value of 0.5 [53,54].

The last step was correlation analysis. The correlation coefficient determines the degree of correlation between the values of two variables [55]. The method of analyzing the correlation of independent variables was used by researchers, including [53]. The Pearson linear correlation coefficient was determined. It is worth noting that the Pearson correlation does not depend on the units of measurement of the analyzed variables [56].

## 3. Test Results

### 3.1. Shore Indentation Hardness Test Results

**Figure 7:**
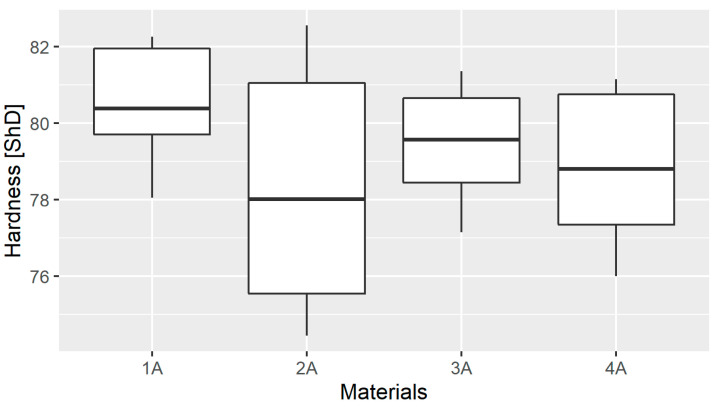
Box plot of hardness in different material groups.

The basic descriptive statistics of the Shore indentation hardness test results obtained in each material group are shown in Table 1 and their graphical presentation in a box plot (Figure 7).

The highest Shore hardness was demonstrated for the 1A material. The measurement results were similar for all materials. Due to small differences in the average Shore hardness values, a statistical test was performed. The selection of an appropriate test (parametric or nonparametric) first requires checking the goodness of fit of the empirical distribution. For this purpose, the S-W test was used. For the tested materials, in each case the test did not give grounds to reject the null hypothesis, all distributions were found to follow a normal distribution (Table 2).

The goodness of fit of the empirical distributions allows parametric tests to be used to examine differences between means in the distribution groups; however, prior to ANOVA, it is still necessary to confirm the equality of variances in the test groups, for which the Levene test was used. The value of the test statistic was W=1.5944, and *p =* 0.2299, indicating that there was no basis for rejecting the null hypothesis of homoscedasticity of variance and allowing ANOVA. The calculated value of the ANOVA test statistic was F=0.755, while *p*=0.535, which means that there is no basis to reject the null hypothesis of the equality of means between groups. Thus, the hardness results obtained for individual materials are not significantly different.

### 3.2. Scratch Test Results

**Figure 8:**
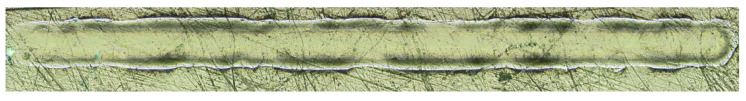
Test scratch on the surface of the 4A material.

Figure 8 shows a crack made with the Rockwell indenter on the surface of the 4A test material. Crack shapes were regular without cohesive damage, i.e., peeling or microcracks [43]. The scratches on the surface of all test materials were similar.

The analysis of scratch hardness results based on the measurements made using the optical microscope (*Hs_mic_*), scratch hardness based on the measurements made using the optical profilometer (*Hs_profil_*), and cross-sectional area of *S_ar_* scratches based on the measurements made using the contact profilometer were further presented.

**Figure 9:**
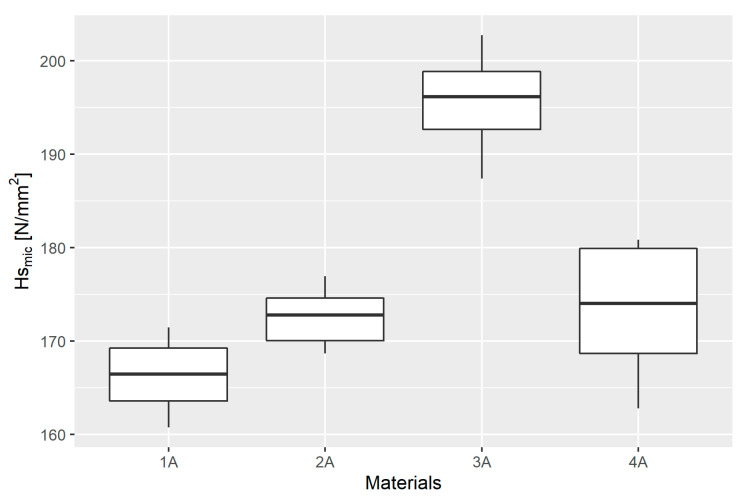
Box plot of scratch hardness results based on the measurements made using the optical microscope (*Hs_mic_*) in individual material groups.

The basic descriptive statistics of the scratch hardness results based on the measurements made using the optical microscope (*Hs_mic_*), obtained in each material group, are presented in Table 3 and their graphical presentation in a box plot (Figure 9).

The goodness of fit was again verified using the S-W test, the results of which are presented in Table 4.

All distributions were found to follow a normal distribution. The Levene test confirmed the homoscedasticity of the variance. The test statistic value was W=1.1442, and p=0.3614. ANOVA was therefore performed. The calculated value of the test statistic was F=26.87, and $p=1.75\cdot 10^{-6}$, which means that the mean values in at least two distributions are significantly different from each other. This was verified in detail using Student’s *t*-test with Bonferroni adjustment, the results of which are presented in Table 5.

The results show the consistency of the means in groups 1A and 2A as well as groups 1A and 4A.

### 3.3. Scratch Hardness

**Figure 10:**
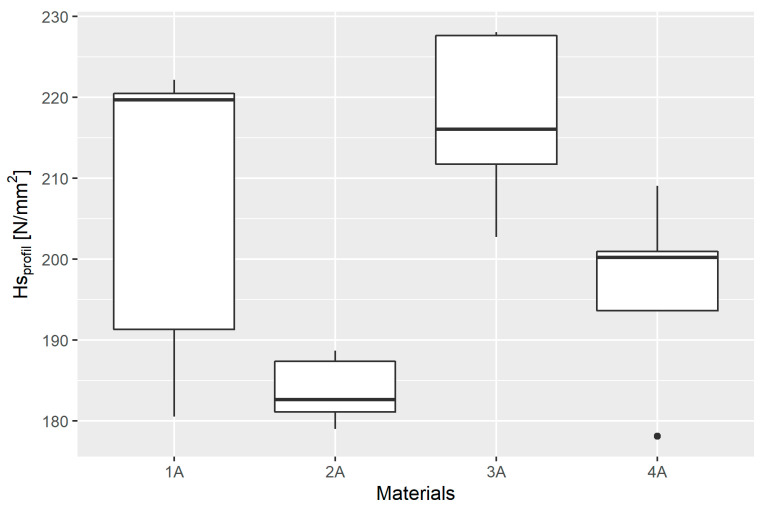
Box plot of the scratch hardness results based on the measurements made using the optical profilometer (*Hs_profil_*) in individual material groups.

The basic descriptive statistics of the scratch hardness results based on the measurements made using the optical profilometer (*Hs_profil_*) obtained in individual material groups are presented in Table 6 and their graphical presentation in a box plot (Figure 10).

The goodness of fit of the empirical distribution was again verified using the S-W test, the results of which are presented in Table 7.

The Levene test confirmed the homoscedasticity of the variance. The test statistic value was W=0.89253, and p=0.4663. ANOVA showed that the mean values in at least two distributions are significantly different from each other. The calculated value of the test statistic was F=6.334, and p=0.0049.

Significantly different groups were determined using Student’s *t*-test with Bonferroni adjustment, the results of which are presented in Table 8.

### 3.4. Scratch Cross-Sectional Area

**Figure 11:**
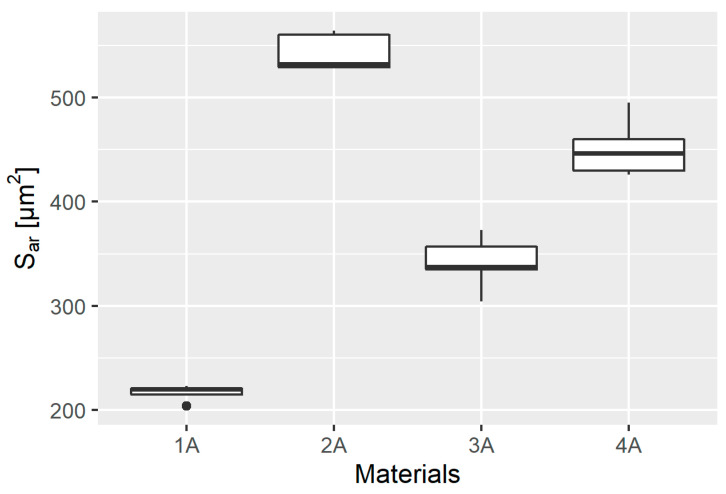
Box plot of measurement of the cross-sectional area of *S_ar_* scratches based on the measurements made using the optical profilometer in individual material groups.

Basic descriptive statistics of the results of measurement of the scratch cross-sectional area of S_ar_ scratches based on the measurements made using the optical profilometer obtained in individual material groups are presented in Table 9 and their graphical presentation in a box plot (Figure 11).

The goodness of fit of the empirical distribution was again verified by means of the S-W test, the results of which are presented in Table 10.

All the distributions were found to follow a normal distribution; therefore, the Levene test was performed. The value of the test statistic was W =0.9994, and p=0.4185, indicating that there was no basis for rejecting the null hypothesis of homoscedasticity of variance and allowing ANOVA. The calculated value of the test statistic was F=218, and $p=3.49\times 10^{-13}$, which means that the mean values in at least two distributions are significantly different from each other. To verify which ones, Student’s *t*-test with Bonferroni adjustment was performed between all pairs of observations, the results of which are presented in Table 11.

The results obtained show that the analyzed means in each of the groups tested are significantly different.

### 3.5. Tribological Wear

**Figure 12:**
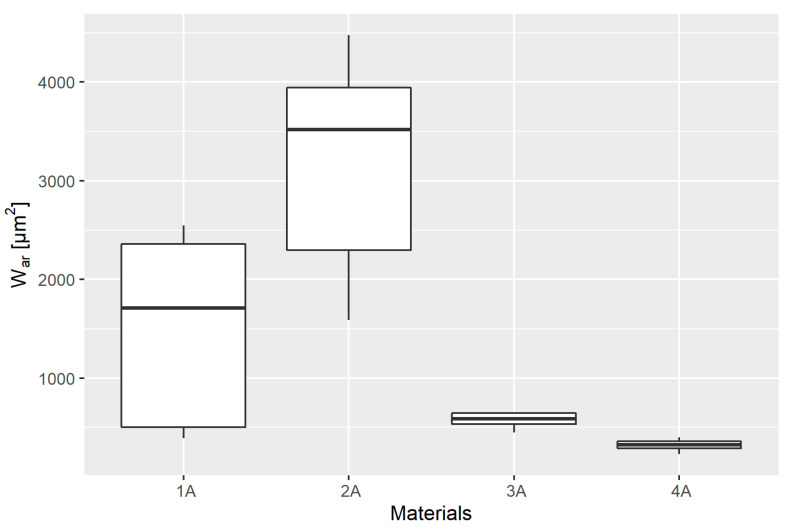
Box plot of tribological wear in individual material groups.

The analysis of wear in each material group also began with the evaluation of basic descriptive statistics presented in Table 12 and the graphical presentation of the results shown in the box plot (Figure 12).

The empirical distribution was then checked for goodness of fit. The results of the S-W test showed the goodness of fit in 2A, 3A, and 4A groups, whereas they did not confirm it in group 1A (Table 13).

In addition, the Levene test (W = 12.32, $p=9.773\times 10^{-06}$) did not confirm the homogeneity of variance; therefore, the non-parametric Kruskal–Wallis test was used to test the differences between the means of the groups of distributions. The value of the test statistic was T=30.659, and $p=1.003\times 10^{-06}$, which means that there are grounds for rejecting the null hypothesis of the equality of means across groups. Thus, the tribological wear results are significantly different for at least two materials. The Wilcoxon rank-sum test was used to verify which groups were different. The results are presented in Table 14.

The above calculations indicate that the groups that differ significantly are 1A and 4A, 2A and 3A, and 3A and 4A.

### 3.6. PCA

In the research in question, we assumed that measurement of hardness using the Shore durometer is the most common and available method among the methods employed in this work for evaluating the surface properties of polymeric materials used for manufacturing orthodontic appliances. This was assumed to be the reference method. In PCA, it was assumed that principal component analysis would identify those measurement methods that provide the most information about the tested materials and therefore could be used in preference to the others. It is intended to facilitate the evaluation of materials in the orthodontist’s laboratory setting. In accordance with the methodology adopted, the first stage of the research was to verify the purposefulness of conducting this analysis using Bartlett’s test and the KMO coefficient. The Bartlett’s test statistic at the significance level of α = 0.05 is $\chi^{2}=33.792$, and p=0.0002. This result allows us to reject the null hypothesis that precludes PCA, which is conditioned by a linear combination of variables. The next step is to evaluate the adequacy of sampling using the Kaiser-Meyer-Olkin (KMO) coefficient. In the case studied, the coefficient KMO=0.5; it therefore takes an acceptable value allowing PCA [57,58,59]. The above results permitted PCA to be conducted and the principal components, which are presented in Table 15 and their properties, presented in Table 16 to be determined.

The cumulative percentage of the explained variance of the analyzed variables was chosen as the selection criterion for reducing the number of principal components (Table 16). According to the literature [52,58,59], one should choose the smallest number of principal components for which the sum of their variances is a certain fraction of the variance of all the variables under reduction. The lower bound that the sum must exceed is, according to various sources, 75, 80, or even 90% [52,58,60].

**Figure 13:**
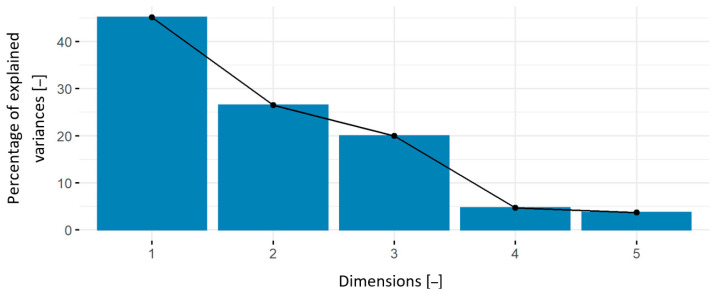
Scree plot.

As can be seen in the plot (Figure 13), when selecting the first component, we get 45% of the explanation of the total variance; when selecting two, −72%; and when selecting three, −91% (Table 16).

The first principal component is the *ShD* measurement, the second one is the *S_ar_* test, and the third one is *Hs_profil_*. These three components explain over 91% of the total variance. The fourth and fifth variables in the scree plot are *Hsmic* and *W_ar_*, respectively. These variables explain the total variance the least. The parameters measured with the use of the contact profilometer explain 46% of the variance. Consideration of profilometric measurements of cross-sectional areas of scratches obtained in the scratch resistance test appears useful in comparisons and rankings of the materials tested in this work. However, given the aforementioned lower bound on the sum of variances, the results of measurements of as many as three parameters are needed to satisfy this bound, but the contribution of *ShD* is the highest.

**Figure 14:**
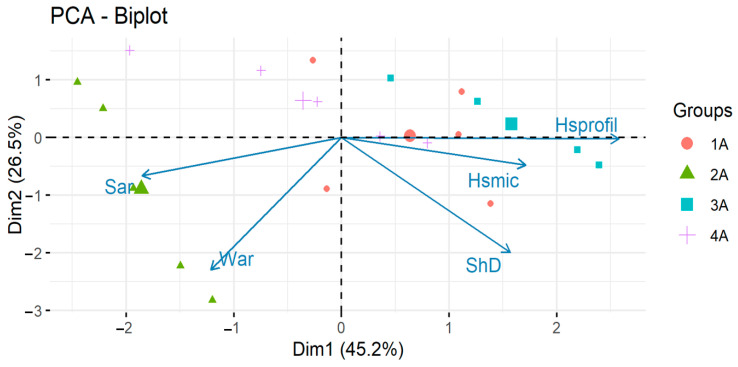
Vectors of original variables.

Figure 14 shows a plot in which the vectors of the original variables and the observaotions representing each case are presented in a coordinate system determined by two principal components.

The length of the arrows approximates the variance of the variables, whereas the angles between them (cosine) approximate their correlations. The acute angles (vectors close to each other) indicate a positive correlation, the right angles (perpendicular vectors) indicate no correlation, and the obtuse angles (vectors on opposite sides of the correlation centre) indicate a negative correlation [52,59].

### 3.7. Analysis of Correlation

**Figure 15:**
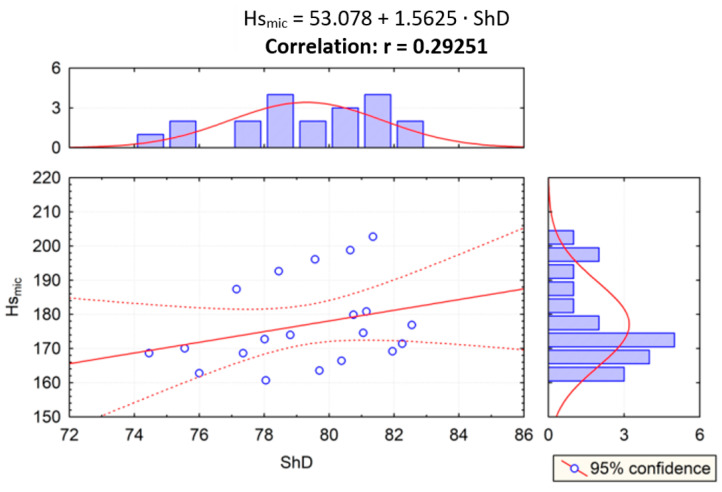
Dependence between scratch hardness *Hs_mic_* and Shore hardness *ShD* (r—correlation).

**Figure 16:**
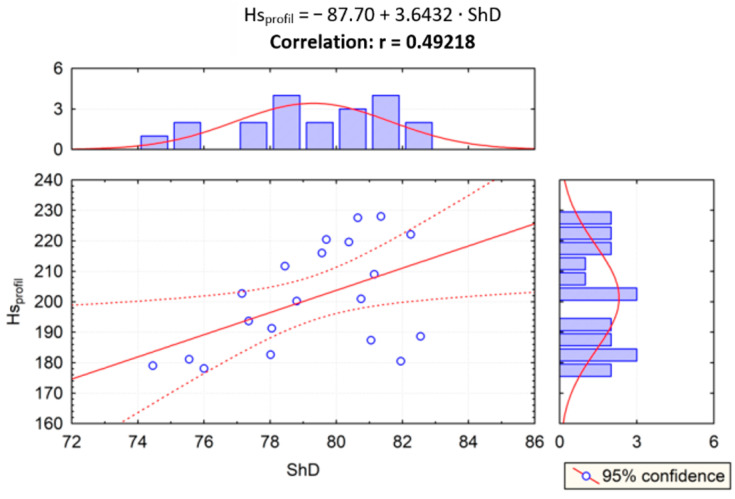
Dependence between scratch hardness Hs_profil_ and Shore hardness ShD (r—correlation).

**Figure 17:**
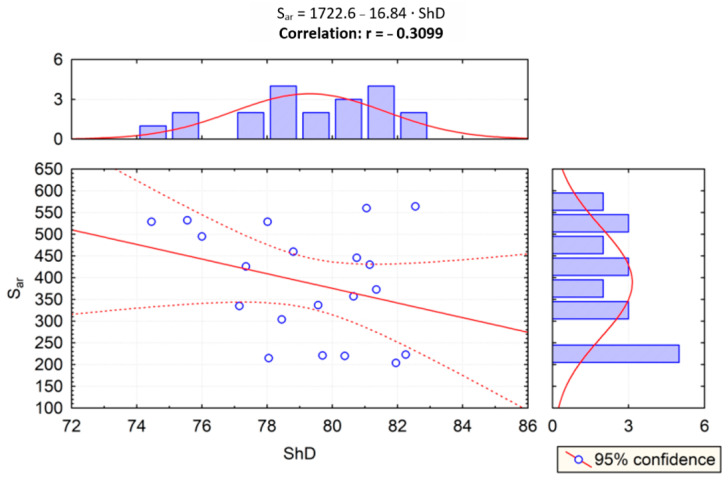
Dependence between cross-sectional area *S_ar_* and Shore hardness *ShD* (r—correlation).

**Figure 18:**
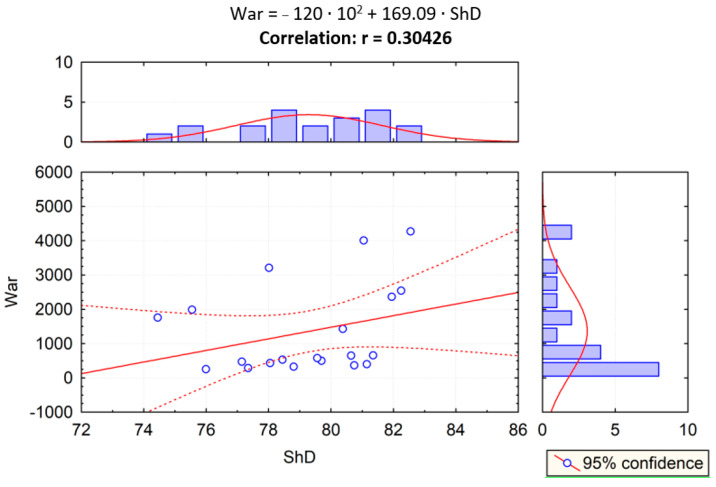
Dependence between cross-sectional area of sliding wear track *W_ar_* and Shore hardness *ShD* (r—correlation).

Given the results of the PCA statistical test, correlation analysis was performed to examine the impact of the relationship of scratch and sliding wear parameters on the Shore hardness, and the correlation between two variables was examined. In Figure 15, the correlation of scratch hardness (*Hsmic*) and Shore hardness (*ShD*) was evaluated. In Figure 15, Figure 16, Figure 17 and Figure 18, the correlation of the other parameters (*Hs_profil_*, *S_ar_*, *W_ar_*) and Shore hardness *ShD* was presented. Furthermore, these figures present regression lines, simple equations, and values of *r* coefficients. The highest level of data concentration around the theoretical (model) line was demonstrated in the case of analyzing the correlation between *Hs_profil_* and *ShD*. The value of the *r* coefficient was in this case the highest and positive, which means that the correlation between these measures is the highest. The highest correlation between *Hs_profil_* and *ShD* indicates that the measurement of the scratch furrow width with the use of the profilometer produces scratch test results that correspond more closely to those of the Shore indentation test—dedicated for polymeric materials. However, this correlation is on the border of moderate and strong correlation according to [61]. The values of the *r* correlation coefficient in the cases of correlation analysis of S_ar_ with *ShD*, *Hsmic* with *ShD*, and *W_ar_* with *ShD* were similar. Such values indicate weak or moderate correlation [61]. In the case of *S_ar_* dependence on *ShD*, a negative correlation was shown, which means that as the value of *ShD* increases, the size of the crack cross section decreases (Figure 17), which, of course, is physically justified. In contrast, the correlation of *W_ar_* and *ShD* is positive. Such a calculation result was obtained because materials 1A and 2A, i.e., the materials having the highest and lowest *ShD*, were characterized by lower and higher sliding wear, respectively. However, for materials 3A and 4A, the relationship was reversed. These materials had the highest wear resistance but were not the hardest in the *ShD* test. They featured, however, the highest *Hs_mic_*.

## 4. Discussion

Bruxism can result in the friction and wear of polymer materials that are used for manufacturing orthodontic appliances. Thegosis is another disease that affects the surface degradation of orthodontic appliances [62]. Bruxism is the action of grinding of teeth without the presence of food, which is regarded as a response to stress and treated clinically as a pathological process [62,63]. Bruxism leads to pathological wear known as attrition, which is not related to the physiological process of food mastication. Attrition is tooth wear in dental terminology. Bruxism treatment should primarily be aimed at eliminating etiological reasons, especially psychological stress. Drug treatment for bruxism is quite promising; however, the use of drugs should take into account the side effects of medicaments [64].

Nowadays, there is no effective method for the treatment of bruxism. The most effective and common treatment option of bruxism is the use of splints. However, the role of splints is limited to protect the teeth from abrasion, as splints do not affect the parafunction of masticatory muscle activity. Given the fact that during normal activity of the stomatognathic system, the period of maximum interdental contact during the day is 10–15 min, in patients with bruxism, this period of time can last 3–4 h, at a masticatory pressure four times above normal. Therefore, the choice of orthodontic appliance design should be approached taking into account the above factors [65]. The process of wear, i.e., attrition, is the most common cause of wear degradation in the dental biotribological system [66]. It occurs as a result of contact and cooperation between opposing teeth [67]. The level of attrition is influenced by occlusal forces and the amount and geometry of wear products [68]. Attrition is a variation of abrasion (wear resulting from cooperation between two bodies). As reported in [69], wear of the enamel surface due to attrition is about 40 µm per year. Hardness of a polymer material is a measure of resistance to concentrated contact forces. Such forces can arise in orthodontic treatment of involuntary tooth clenching. The action of these forces can lead to permanent damage through local deformation of a material used for orthodontic appliances where concentrated biomechanical forces act, e.g., due to contact with canine cusps [70]. Orthodontic appliances that serve to prevent the effects of these diseases should take over these pathological forces and movements to relieve pressure on teeth without a loss of functionality when used by a patient. At the same time, orthodontic appliances are susceptible to damage of their surface. Researchers have confirmed that the quantity determining the functional quality of orthodontic appliances under these loading conditions is hardness [18]. Undoubtedly, wear resistance is an equally important feature [9]. In general, wear depends on the hardness of the materials in the friction node, on the load, and on the friction path. The shape and protruding irregularities of the couple’s harder material act as microblades. Wear is caused by micro-cutting, scratching, and furrowing. Wear products in the friction area also influence the degree of wear [71]. In clinical situations, damage to polymeric orthodontic appliances can be scratch damage furrows. Unfortunately, it has been shown that most polymer materials are prone to scratch damage [32]. Such surface damage can be caused by sharp irregularities at the edge of teeth. Such irregularities can be created by, e.g., chipping of enamel at the incisor edge in the disease process. Another cause of irregularities is inaccurate enamel preparation and cavity filling. Such inaccurate preparation contributes to the formation of irregularities along the line of the filling [31,67]. When the surface of one body has a higher hardness than the antagonistic one, as it is in the analyzed case, there is rapid wear of the surface by micro-scratching [72]. Scratching in the friction direction is evidence of abrasion wear of polymers [73], so the scratching mechanism is related to sliding wear. It was more important in this work, however, to determine to what extent scratch resistance correlates with Shore indentation hardness due to the greater proliferation and practical use of the Shore indentation method. Hardness testing is comparatively simple, quick, and efficient [74], Some researchers point out that scratch hardness testing is a method that gives different results than indentation hardness testing [32]. However, in [75], an analysis of correlation of indentation hardness according to Oliver and Pharr’s method and scratch hardness of polymeric materials, including PMMA, was undertaken. The authors stated that if the testing conditions are consistent, scratch hardness and indentation hardness appear to be quite similar for almost all materials. At the present stage, this is a purely phenomenological observation [75], which partly justifies the correlation dependencies presented in this paper. In [75], the authors also stated that the indentation hardness according to Oliver and Pharr’s (O&P) method depends on recovery characteristics from the unloading curve. In the case of some polymer materials, a large proportion of elastic deformation under the influence of the indenter has a significant impact on the indentation hardness according to the O&P method. However, in the scratch hardness test, the effect of recovery characteristics of the material is not as obvious as in the case of indentation [75]. However, this phenomenon has not been demonstrated in the indentation tests of PMMA, which was the subject of research in this article. It has been shown for melamine–formaldehyde polymer (MF). It is probably related to the fact that the penetration depth during the indentation of MF is small. However, for other polymers, including PMMA, the penetration depth is large enough to ensure contact mainly with the conical part of the Rockwell indenter [75]. It is possible that when testing with the Shore D indenter, which is tapered and which also has a rounded tip of the cone, similar phenomena as when using the Rockwell indenter may occur. This relationship was also confirmed in [76].

The highest statistical compatibility of *Hs_profil_* from the scratch test with the Shore indentation test, which is commonly used in the evaluation of polymer materials, indicates the usefulness of *Hs_profil_* in comparative tests and the creation of a ranking of the surface layer properties of materials. The *Hs_profil_* measure allows to capture the impact of plastic pile-up flow under the action of the indenter to a greater extent than *Hs_mic_*, which varies for polymer materials [77]. In contrast, the size of *S_ar_* is the measure that best describes the surface damage profile [40] and allows us to capture the pile-up deformation and elastic recovery of polymeric materials (this phenomenon mainly affects the side walls of the crack furrow). From a practical (engineering) point of view and given the normative requirements [78], the *SW_mic_* measure, determined using an optical microscope, which is the quantity needed to calculate *Hs_mic_*, should be used. However, in scientific studies of the contact strength of the surface layer of polymer materials, one should also consider the use of values obtained with the use of a contact profilometer. The values of *War* and the *ShD* correlation coefficient are positive and similar to those obtained in the scratch analysis using the contact profilometer. The evaluation of the correlation strength shows a low correlation and a clear relationship. Compared to metals, there are only a few theoretical models for the wear of polymer and polymer composites, and there are no universal governing theories [79]. In [80], the wear rate equation of polymer plastics contains the indentation hardness. In addition, in much earlier papers [81,82,83], an equation for the wear of polymers containing indentation hardness has been given. However, some papers, such as [84], present wear equations independent of hardness. In analyzing the test results, it should also be taken into account that sliding wear tests were conducted under wet conditions in an artificial saliva bath at a temperature of 37 °C. The tribological behavior of polymers under lubricated conditions may significantly differ from dry contacts, due to the effects arising from the presence of a lubricant [76]. Artificial saliva in the present situation acts as a lubricating medium. It seems that at least one mechanism—hydrodynamic lubrication—may be relevant in the situation described. Since polymers, in general, have relatively low elastic modulus, hydrodynamic effects can become significant at much lower speeds than in the case of metals [76]. However, the obtained wear indicates that the kinematic couple friction conditions adopted according to [69], characterized by a low frequency corresponding to the mandibular movement frequency, may have influenced the occurrence of mixed and boundary lubrication. Testing in artificial saliva can also lead to hydrolytic degradation, chiefly deteriorating surfaces of polymer material samples being in contact with liquid. Saliva is of physiological importance and is constantly present in the human mouth. Saliva is in contact with the surfaces of solids in the mouth. They are covered with a layer of absorbed saliva proteins and acquired pellicle. Such a coating is formed after a few seconds in the mouth on any hard surface. [69,85]. It has been found that amorphous polymers are more prone to hydrolytic degradation than crystalline ones and linear polymers than branched ones, polymers of higher molecular weight. Degradation depends on the presence of specific chemical groups in the molecule, including ester, amide, and urea groups [86]. The intensity of degradation also depends on the condition of material surfaces, defects in the material, and the type and percentage of additional substances [86,87]. The synergistic interaction of the described factors translates into a low correlation between *W_ar_* and *ShD*. In light of the results of PCA, in which *W_ar_* was shown to explain only 4% of the total variance in test results, and given the directions of the vectors of these variables, which are shown in Figure 11, it can be concluded that *ShD* is a measure with little dependence on *W_ar_*. The ranking of the sliding wear resistance of the tested orthodontic materials should be made separately.

Effective use of *ShD* as a useful measure for ranking orthodontic acrylic materials can only be limited. Inference from differences in Shore hardness can be difficult because differences in Shore hardness may be negligible for similar materials with identical purpose. An example is the paper on the modification of PMMA for the denture base [88]. The authors of this interesting paper showed the impact of material modification on a number of mechanical properties but not in the case of *ShD*. They claimed that some differences in Shore hardness are shown but they are insufficient to evaluate the differences, which can be influenced by various factors, including surface porosity and residual monomer concentration.

## 5. Conclusions

Based on the literature study and our own research, the following conclusions were made:

1. Shore indentation hardness and scratch hardness are two different quantities that enable identification of the mechanical properties of the surface layer of polymer materials. Since both hardness measures reflect clinical load cases, they can be useful in assessing the performance of orthodontic biomedical polymer materials.
2. This research showed greater variation in the scratch hardness of the tested materials than in the Shore indentation hardness. It seems that the scratch hardness method allows one to create a more explicit ranking of orthodontic polymer materials.
3. The results obtained indicate that scratch hardness measurements are only partially representative of Shore indentation hardness. The highest correlation between scratch hardness and Shore indentation hardness was found in the case of scratch hardness measurements using the profilometer.
4. For the orthodontic materials tested, *ShD* was shown to be a measure with little dependence on *W_ar_*. The evaluation of the correlation strength shows a low correlation. The ranking of the sliding wear resistance of the tested orthodontic materials should be made separately, not based only on the Shore hardness.

## Figures and Tables

**Table 1:** Basic descriptive statistics of hardness measurement results in individual material groups.

Material	Number of Measurements	Mean	Median	SD	Min.	Max.
1A	5	80.47	80.38	1.72	78.05	82.25
2A	5	78.32	78.01	3.47	74.45	82.55
3A	5	79.43	79.56	1.68	77.15	81.35
4A	5	78.81	78.81	2.19	76.00	81.15

**Table 2:** S-W test results for Shore indentation hardness test values for individual materials.

Material	W-Test Statistics Value	*p*-Value
1A	0.94102	0.6731
2A	0.93657	0.6418
3A	0.97243	0.8906
4A	0.93463	0.6283

**Table 3:** Basic descriptive statistics of the scratch hardness results based on the measurements made using the optical microscope (*Hs_mic_*) in individual material groups.

Material	Number of Measurements	Mean	Median	SD	Min.	Max.
1A	5	166.29	166.45	4.29	160.73	171.46
2A	5	172.61	172.77	3.34	168.68	176.93
3A	5	195.55	196.12	5.87	187.40	202.74
4A	5	173.24	174.01	7.62	162.80	180.83

**Table 4:** S-W test results for individual materials.

Material	W-Test Statistics Value	*p*-Value
1A	0.97978	0.9335
2A	0.97068	0.8796
3A	0.99262	0.988
4A	0.92852	0.5863

**Table 5:** Results of Student’s *t*-test with Bonferroni adjustment.

Group 1	Group 2	n1	n2	*p*-Value	Adjusted *p*-Value
1A	2A	5	5	0.089	0.537
1A	3A	5	5	0.000306	0.00000183
2A	3A	5	5	0.0000065	0.000039
1A	4A	5	5	0.0639	0.384
2A	4A	5	5	0.857	1
3A	4A	5	5	0.00000907	0.0000544

**Table 6:** Basic descriptive statistics of the scratch hardness results based on the measurements made using the optical profilometer (*Hs_profil_*) in individual material groups.

Material	Number of Measurements	Mean	Median	SD	Min.	Max.
1A	5	206.814	219.65	19.479	180.52	222.12
2A	5	183.78	182.67	4.13	179.01	188.69
3A	5	217.23	216.02	10.80	202.74	228.04
4A	5	196.40	200.24	11.58	178.11	209.04

**Table 7:** S-W test results for individual materials.

Material	W-Test Statistics Value	*p*-Value
1A	0.793	0.07097
2A	0.82576	0.5678
3A	0.91439	0.4944
4A	0.92708	0.5766

**Table 8:** Results of Student’s *t*-test with Bonferroni adjustment.

Group 1	Group 2	n1	n2	*p*-Value	Adjusted *p*-Value
1A	2A	5	5	0.0113	0.0676
1A	3A	5	5	0.214	1
2A	3A	5	5	0.000741	0.00445
1A	4A	5	5	0.214	1
2A	4A	5	5	0.136	0.818
3A	4A	5	5	0.0198	0.119

Distributions 2A and 3A are the only ones that show significant differences in group means.

**Table 9:** Basic descriptive statistics of the results of measurement of the cross-sectional area of *S_ar_* scratches based on the measurements made using the optical profilometer in individual material groups.

Material	Number of Measurements	Mean	Median	SD	Min.	Max.
1A	5	216.60	220.00	7.64	204.00	223.00
2A	5	542.80	532.00	17.63	529.00	564.00
3A	5	341.20	337.00	25.98	304.00	373.00
4A	5	451.40	446.00	27.87	426.00	495.00

**Table 10:** S-W test results for individual materials.

Material	W-Test Statistics Value	*p*-Value
1A	0.84967	0.1935
2A	0.76056	0.03717
3A	0.96948	0.8719
4A	0.90748	0.452

**Table 11:** Results of Student’s *t*-test with Bonferroni adjustment.

Group 1	Group 2	n1	n2	*p*-Value	Adjusted *p*-Value
1A	2A	5	5	5.06 · $10^{-14}$	3.04 $\cdot \;10^{-13}$
1A	3A	5	5	8.23 · $10^{-8}$	4.94 $\cdot 10^{-7}$
2A	3A	5	5	8.12 · $10^{-11}$	4.87 $\cdot 10^{-10}$
1A	4A	5	5	8.10 · $10^{-12}$	4.86$\;\cdot 10^{-11}$
2A	4A	5	5	4.47 · $10^{-6}$	2.68 $\cdot \;10^{-5}$
3A	4A	5	5	4.24 · $10^{-7}$	2.55 $\cdot \;10^{-6}$

**Table 12:** Basic descriptive statistics of tribological wear measurement results in individual material groups.

Material	Number of Measurements	Mean	Median	SD	Min.	Max.
1A	10	1452.58	1425.91	996.57	431.00	2541.00
2A	10	3207.80	3520.00	1017.98	1589.00	4477.00
3A	10	575.56	588.30	74.40	447.50	654.01
4A	10	325.90	325.00	55.21	228.50	401.00

**Table 13:** S-W test results for individual materials.

Material	W-Test Statistics Value	*p*-Value
1A	0.79665	0.0085
2A	0.88749	0.1589
3A	0.90129	0.2264
4A	0.94621	0.6239

**Table 14:** Wilcoxon rank-sum test results for tribological wear in individual groups.

	1A	2A	3A
2A	0.0033		
3A	0.3979	2.2$\;\cdot 10^{-5}$	
4A	3.4$\;\cdot 10^{-5}$	2.2$\;\cdot 10^{-5}$	2.2$\;\cdot 10^{-5}$

**Table 15:** Principal components (PC).

	PC1	PC2	PC3	PC4	PC5
*ShD*	0.381	−0.633	0.231	−0.532	−0.343
*Sar*	−0.451	−0.211	−0.637	−0.523	0.270
*Hsprofil*	0.626	−0.009	−0.057	−0.066	0.775
*Hsmic*	0.416	−0.152	−0.721	0.397	−0.357
*War*	−0.295	−0.729	0.135	0.531	0.285

**Table 16:** Components’ significance.

	PC1	PC2	PC3	PC4	PC5
Standard deviation	1.503	1.151	0.999	0.484	0.429
Proportion of variance	0.452	0.265	0.200	0.047	0.037
Cumulative proportion	0.452	0.717	0.916	0.963	1.000

## Data Availability

Data sharing is not applicable to this article.
